# New-Onset Seizure Following Loratadine and Alcohol Ingestion: A Case Report

**DOI:** 10.7759/cureus.112779

**Published:** 2026-07-16

**Authors:** Morgan Denecke, Thomas Stack, Jackson Beall, Joshua Oliver

**Affiliations:** 1 Emergency Medicine, Madigan Army Medical Center, Tacoma, USA; 2 Emergency Medicine, Edward Via College of Osteopathic Medicine, Blacksburg, USA; 3 Arts and Sciences, University of Washington, Seattle, USA

**Keywords:** anti-histamine, emergency medicine, seizure, toxicology, toxidrome

## Abstract

Loratadine is a common over-the-counter second-generation H1-antihistamine used to relieve and treat seasonal allergy symptoms. While first-generation H1-antihistamines are historically known to cause central nervous system (CNS) side effects, these effects are thought to be far less common in second-generation antihistamines. Similarly, alcohol is known to have CNS effects. We report a case of a 26-year-old female who presented to the emergency department (ED) after a suicide attempt involving loratadine and alcohol ingestion. Emergency medical services (EMS) reported that the patient became unresponsive upon arrival at the ED. An empty loratadine bottle that was supposed to contain 60 tablets was found at the scene. Additionally, an empty bottle of wine was also found at the scene. Initial vital signs and glucose levels were normal, but on examination, the patient had a Glasgow Coma Scale of 3 with rightward eye deviation and developed tonic-clonic seizures, which were quickly controlled with 4 mg of intravenous (IV) lorazepam. She gradually regained responsiveness and received 3200 mg of levetiracetam IV. Toxicological workup revealed a serum ethanol level of 119 mg/dL and a urine toxicology screen positive for benzodiazepines, likely due to lorazepam administration. The patient had mildly elevated lactate and creatine kinase levels consistent with seizure, which normalized after fluid resuscitation. The patient recovered fully without further seizures and was not prescribed antiepileptic medication upon discharge. We present the case to document a potential seizure provoked by the combined ingestion of loratadine and alcohol.

## Introduction

One in three adults in the United States suffers from seasonal allergies [1]. Loratadine is one of many widely used over-the-counter second-generation antihistamines for symptom relief of seasonal allergies, as well as the treatment of symptoms of allergic reactions [2]. First-generation H1-antihistamines such as diphenhydramine have poor selectivity and are notable for their ability to cross the blood-brain barrier, which can cause significant central nervous system (CNS) suppression and seizures [3]. These side effects led to the development of second-generation H1-antihistamines in the 1980s [3]. They have reduced penetration across the blood-brain barrier compared to first-generation and have no anticholinergic effects due to their high selectivity for the histamine H1 receptor [2].

There is limited evidence that suggests that histamine plays an important role in inhibiting seizures through the activity of the H1 receptors [4]. Second-generation antihistamine pharmacodynamics are well studied and are understood to have less CNS penetrance than first-generation, although they still achieve detectable levels [5]. However, loratadine has been found to potentially cause CNS depression when the manufacturer’s dose is exceeded, supporting the assertion that these medications bind at levels in the CNS that can cause side effects [5].

While second-generation antihistamines are generally not thought of as a seizure-inducing medication, cases have been documented in which desloratadine, a second-generation antihistamine, has induced absence seizures in pediatric populations [4]. However, we were unable to find cases of loratadine overuse provoking a seizure in an adult [6].

According to the American Heart Association, approximately 85% of Americans report alcohol consumption at some point in their life, making it a commonly consumed substance [7]. Original studies into the relationship between alcohol consumption and seizures suggested a possible relationship with first-time seizures. A case-control study published in 1988 demonstrated that sixteen percent of first-time seizures in persons consuming alcohol could not be attributed to alcohol withdrawal seizures [8]. This led them to conclude that there was a causal relationship between alcohol consumption and seizures [8]. However, recent evidence has made this proposed relationship less clear. A 2022 meta-analysis from Woo et al. showed that among the 8 examined studies, there was an increased risk for seizure amongst alcohol consumers [9]. On further analysis, the five examined case-control studies demonstrated this relationship, whereas the three cohort studies demonstrated no relationship between alcohol use and first-time seizures [9]. Additional research on patients with epilepsy does suggest that social drinking does not increase the incidence of seizures, but binge drinking does [10].

While alcohol’s ability to provoke seizures is unclear, there are several proposed mechanisms. Animal models have been utilized to demonstrate two different mechanisms. Low concentrations of alcohol in rat hippocampal tissue were shown to decrease adenosine release, resulting in increased electrical activity [11]. A second study utilizing a mouse model demonstrated that acute alcohol ingestion induced microglial activation in the mouse hippocampus, which ultimately resulted in enhanced seizure-like activity [12]. There is overall mixed evidence regarding the roles of both second-generation antihistamines and alcohol in the primary provocation of seizures.

Furthermore, there do not appear to be synergistic effects between the two drugs. Alcohol is primarily metabolized in the liver by CYP2E1, whereas loratadine is metabolized in the liver by CYP3A4 and CYP2D6 [13,14].

## Case presentation

The patient was a 26-year-old female with no relevant past medical history or prior diagnosis of seasonal allergies who presented to the emergency department via emergency medical services (EMS) following a suicide attempt. Upon arrival in the ED, she was unresponsive with a Glasgow Coma Scale (GCS) score of 3. EMS noted that she had been awake and conversant but became unresponsive just prior to arrival. They reported that the patient had ingested an unknown amount of over-the-counter loratadine. An empty bottle that had originally contained 60 tablets, as well as an empty bottle of wine, was found at the scene.

The initial set of vital signs was as follows: heart rate of 105 beats per minute, respiratory rate of 14 breaths per minute, blood pressure of 139/98 mmHg, oxygen saturation of 100% on room air, temperature of 36.7°C, and point-of-care glucose of 95 mg/dL (reference range: 74-109 mg/dL). The eye examination was notable for disorganized eye movements with right lateral gaze deviation. She developed generalized tonic-clonic movements, which responded to 4 mg of intravenous lorazepam. Over the next 20 minutes, the patient gradually became more responsive and was loaded with 3200 mg of IV levetiracetam. A full workup for altered mental status and toxic ingestion was obtained to include a complete blood count, complete metabolic panel, pregnancy test, venous blood gas, creatine kinase, lactic acid, urinalysis, urine drug screen, salicylate level, acetaminophen level, head CT, and EKG. This revealed only an ethanol level of 119 mg/dL and a positive result for benzodiazepines, which was likely related to the administration of lorazepam. Otherwise, the laboratory evaluation showed a mildly elevated lactate to 2.4 mmol/L (reference range: 0.5-2.2 mmol/L) and creatine kinase to 283 U/L (reference range: 26-192 U/L), which improved after fluid resuscitation with 1 liter of normal saline over 1 hour. Liver enzymes were within the reference range. All other laboratory results were within the reference range, and no metabolic derangements were noted. An EKG did not show evidence of arrhythmia or interval prolongation. A head CT did not show any acute process or other abnormalities. We consulted Poison Control, which recommended maintenance of electrolyte levels within normal limits but stated that toxic effects, including seizures, were atypical of loratadine overdoses.

Once conversant, the patient reported no history of seizures. She reported ingesting an unknown amount of loratadine and alcohol. The loratadine bottle, which was brought to the ED, had originally contained sixty 10-mg tablets

She was ultimately admitted to the medicine service for continued observation and workup of the seizure. She was evaluated by Neurology, which suspected that the patient likely experienced a seizure due to loratadine overuse. An electroencephalogram performed during her admission did not show evidence of seizure activity. At the neurologist's recommendation, the patient was discharged without a prescription for antiepileptic medication. Chart review revealed that the patient had made a complete recovery without additional seizures.

## Discussion

We present this case to highlight what we believe to be an extremely rare side effect of the combined ingestion of loratadine and alcohol. The patient's clinical presentation and laboratory findings were consistent with a seizure. Based on our discussion with the patient, we suspect that the etiology of the seizure was an overdose of loratadine with concurrent alcohol ingestion. The neurologist who evaluated the patient also suspected that she likely experienced a seizure secondary to loratadine ingestion. However, it is difficult to disentangle the effects of the two substances and directly attribute the seizure to one substance.

As with any suspected toxicologic case, the initial evaluation of the patient should be broad and include typical laboratory evaluation of suspected overdose. This workup can include a complete metabolic panel, drug screen, salicylate level, acetaminophen level, pregnancy test for females, as well as additional labs deemed suitable by the provider [15]. A venous blood gas and serum osmolality may be helpful in some scenarios in which the intoxicant is not clear [16]. An EKG, continuous cardiac monitoring, and pulse oximetry are indicated in patients with an antihistamine overdose, as they are at risk for arrhythmias, including torsades de pointes, due to QT prolongation [17]. Furthermore, a full set of vitals, including point-of-care glucose and temperature, must also be obtained to monitor for side effects of intoxicants.

Management of antihistamine ingestion is directed toward the typical steps used in other toxicologic cases. Activated charcoal can be utilized within the first hour after ingestion if the patient is alert and oriented and able to participate in consumption of the medication. Resulting wide-complex tachyarrhythmias are managed with the administration of sodium bicarbonate because, in high quantities, antihistamines act as sodium channel blockers, and the sodium in sodium bicarbonate can overwhelm that blockade [17]. Frequently, patients presenting with antihistamine overuse will present with severe agitation, which can be managed by the administration of benzodiazepines [18]. Patients' symptoms of agitation can be managed with the use of lorazepam 1-2 mg or midazolam 2.5-5 mg as needed.

Although we were unable to find guidelines regarding the treatment of mixed loratadine- and alcohol-induced seizures, management can be extrapolated from the treatment of seizures induced by first-generation antihistamines. When first-generation antihistamines provoke seizures, they can be managed with benzodiazepines [19]. Typically, administration of 2-4 mg of lorazepam is used as the first-line therapy, with increased doses as needed [19]. In this case, the patient was managed with 4 mg of lorazepam, which resulted in termination of the seizure. However, if the patient had not responded, an appropriate second-line agent for status epilepticus, such as levetiracetam, would have been utilized. This would be followed by intubation with propofol or ketamine to further treat the patient's seizure and to protect the patient's airway in the setting of increased doses of sedating medications.

During the initial management in the ED, the patient was also loaded with 60 mg/kg of levetiracetam due to the initially unclear etiology of the patient's seizures. Ultimately, per the neurologist's recommendation, the patient was not started on additional antiepileptic medications prior to discharge from the hospital.

## Conclusions

We present this case to highlight what may be a potentially uncommon provocation of seizures. The patient presented to the emergency department shortly after ingestion of an unspecified amount of loratadine and alcohol. In this instance, we demonstrated seizure termination through the use of benzodiazepines. Additionally, there was no seizure recurrence noted on EEG or in the outpatient setting. We believe it is vitally important that emergency medicine physicians are aware that excessive loratadine, when used in conjunction with alcohol, can potentially provoke seizures. We hope that physicians can learn from our experience managing such a rare case.
